# Modulatory Effects of Guarana (*Paullinia cupana*) on Adipogenesis

**DOI:** 10.3390/nu9060635

**Published:** 2017-06-20

**Authors:** Natália da Silva Lima, Erica de Paula Numata, Leonardo Mendes de Souza Mesquita, Pollyana Hammoud Dias, Wagner Vilegas, Alessandra Gambero, Marcelo Lima Ribeiro

**Affiliations:** 1 Laboratory of Immunopharmacology and Molecular Biology, Clinical Pharmacology and Gastroenterology Unit, Sao Francisco University Medical School, Braganca Paulista-SP 12916-900, Brazil; aykime@hotmail.com (E.d.P.N.); alessandra.gambero@usf.edu.br (A.G.); 2 Laboratory of Bioprospection and Natural products (LBPN), UNESP-São Paulo State University/Coastal Campus of Sao Vicente, Pça Infante Dom Henrique S/N, São Vicente, São Paulo 11330-900, Brazil; mesquitalms@gmail.com (L.M.d.S.M); pollyhdias@gmail.com (P.H.D.); vilegasw@gmail.com (W.V.)

**Keywords:** adipogenesis, guarana (*Paullinia cupana*), obesity, 3T3L1, Wnt pathway, miRNA

## Abstract

Guarana (*Paullinia cupana*) is a plant originated in Brazil that presents a beneficial effect on body weight control and metabolic alterations. The aim of this study was to evaluate the effects of guarana on genes and miRNAs related to adipogenesis in 3T3L1 cells. The anti-adipogenic effect of guarana was evaluated by Oil Red-O staining. Gene and miRNA expression levels were determined by real time PCR. The Cebpα and β-catenin nuclear translocation were evaluated using immunocytochemistry. Our data indicated that the triglyceride-reducing effect of guarana was dose-dependent from 100 to 300 µg/mL (−12%, −20%, −24% and −40%, respectively, *p* < 0.0001). An up-regulation of the anti-adipogenic genes *Wnt10b*, *Wnt3a*, *Wnt1*, *Gata3* and *Dlk1* and a down-regulation of pro-adipogenic genes *Cebpα*, *Pparγ* and *Creb1* were also observed. Furthermore, guarana repressed mmu-miR-27b-3p, mmu-miR-34b-5p and mmu-miR-760-5p, that contributed for up-regulation of their molecular targets *Wnt3a*, *Wnt1* and *Wnt10b*. Additionally, cells treated with guarana presented an increase on β-catenin nuclear translocation (*p* < 0.0018). In summary, our data indicate that guarana has an anti-adipogenic potential due to its ability to modulate miRNAs and genes related to this process. Together our data demonstrate the important role of guarana as a putative therapeutic agent.

## 1. Introduction

Obesity is a non-communicable disease that affects distinct age groups, ethnic groups and social classes. It has recently shown that obesity affects 13% of the worldwide population [1]. Although there are healthy obese subjects [2], it has already been demonstrated that the risk of development of ischemic heart disease is irrespective of metabolic status [3]. Metabolically unhealthy obesity is more common and is characterized by exacerbated increase of adipose tissue that is dependent on intense and complex cellular remodeling [2]. The activation of several transcriptional factors is necessary for adipogenesis. Among them, we highlight *Cebpα* and *Pparγ*; these are the two most important genes involved in this process [4]. Furthermore, it has been demonstrated that miRNAs play crucial roles in many physiological and pathological processes due to their capacity to modulate several important genes [5], including genes related to adipogenesis [6]. Considering the great relevance of this issue, several studies have evaluated the influence of bioactive compounds on obesity and other associated diseases.

Guarana (*Paullinia cupana*) is a plant originated in Brazil, rich in several bioactive compounds such as methylxanthines (including caffeine, theobromine and theophylline) tannins, saponins, catechins, epicatechins and proanthocyanins [7]. Guarana has been associated with protection against hypertension, obesity and metabolic syndrome in elderly healthy volunteers [8]. Additionally, a therapeutic role in atherosclerosis has been described [9]. Complementarily, the association of yerba mate, guarana and damiana (“YGD”) was associated with lower food intake in overweight woman [10]. An in vitro study demonstrated that the “YGD” association was able to reduce the expression of pro-adipogenic genes such as *Cebpα*, *Adig*, *Pparγ* and increase expression of anti-adipogenic genes such as *Klf2* and *Ucp1* [11]. 

Although some in vitro [11] and human studies [8,10] indicate the anti-adipogenic potential of guarana, its effects on adipogenesis have not been evaluated. Here, we are able to show that guarana exerts its anti-adipogenic activity through the modulation of both: the expression of master regulators of adipogenesis, and the activation of the Wnt pathway.

## 2. Materials and Methods

### 2.1. 3T3L1 Cell Culture

The 3T3-L1 cell line was purchased from the Rio de Janeiro Cell Bank (Rio de Janeiro, Brazil) and it was cultured to confluence in Dulbecco’s modified Eagle’s medium (DMEM) supplemented with 10% fetal bovine serum (FBS) and 1% of penicillin/streptomycin/glutamin at 37 °C and 5% CO_2_.

### 2.2. Cytotoxicity and Triacylglycerol Accumulation

The determination of cell toxicity and triacylglycerol accumulation were performed using the following guarana concentrations (50, 100, 150, 200 and 300 µg/mL). For this, tetrazolium salt reduction test (MTT) and Oil Red O methods previously described were used [11].

### 2.3. Experimental Design

Forty-eight hours after achieving confluence (day 0), cells were divided in two groups: Control—incubated in differentiation medium (DMEM supplemented with 10% fetal bovine serum, 1% of penicillin/streptomycin/glutamine, 0.01% of dexamethasone (1 mM), 0.01% of insulin (100 UI) and 0.1% of IBMX (0.5 mM)) and Guarana—incubated in differentiation medium + guarana (chosen dose: 150 μg/mL). For each group, six replicates were realized. After 96 h of incubation (4 days) cells were collected and stored at −80 °C.

### 2.4. Total Phenolic Content

The determination of total phenolics content was performed using the Folin-Ciocalteu reagent with some modifications [12].

### 2.5. Total Flavonoid Content

Flavonoid content determination was carried out using the colorimetric method in which the complexation of flavonoids with aluminum chloride occurs according to the adapted standard methodology [13].

### 2.6. Caffeine Quantification by Mass Spectrometry

Caffeine quantification was performed using the Waters^®^ Acquity UPLC system (Waters Corp., Milford, MA, USA) consisting of a quaternary pump, autosampler, and mass spectrometer equipped with XevoTqD^®^ (Waters Corporation, Milford, MA, USA) source of electro-spray ionization (ESI) and analyzer triple-quadrupole. The source parameters were set as follows: desolvation gas flow 350 L/h, desolvation temperature 120 °C, collision gas flow 1 L/h and source temperature 150 °C. Capillary voltage was set to 3.30 V and the cone voltage was set to 30 V. Caffeine detection was performed using multiple reaction monitoring mode (MRM), the transition 195 > 138 for measurement. Quantification was performed through the external standard method, with a caffeine calibration curve with concentrations of 1.0, 1.5, 2.0, 3.0, 3.5, and 5.0 ppm, injected in triplicate in linear regression. It was possible to obtain the value of the correlation coefficient (*r*^2^) of 0.99%. The value found for the sample was extrapolated from the calibration curve to obtain the caffeine concentration of guarana. All analyzes were performed using MassLynx software (Waters Corporation).

### 2.7. mRNA and miRNA Expression

Total RNA extraction, cDNA synthesis and quantitative PCR were performed as previously described [11], using specific primers (Table 1). Real-time PCR was performed in a 7500 real-time PCR system (Applied Biosystems, Foster City, CA, USA). The expression of 18S and β-actin were used as endogenous control for data normalization. The results were analyzed using the 2^−ΔΔ*C*t^ relative quantification method.

The miRNA extraction was performed using miRNeasy Mini Kit (QIAGEN, Valencia, CA, USA). After the extraction, the RNA was reverse-transcribed using the miScript II RT Kit (QIAGEN) following the manufacturer’s protocol. The samples were subjected to TaqMan miRNA assays for mmu-miR-27b-3p, mmu-miR-34b-5p and mmu-miR760-5p expressions (ID: 000409, 002617 and 463135_mat, respectively, Applied Biosystems) according to the manufacturer’s instructions. Real-time PCR was performed on the resulting cDNA using miR specific TaqMan primers and TaqMan Universal PCR Master Mix in a 7500 real-time PCR system (Applied Biosystems). The expression of mmu-miR-U6 (TaqMan primers, ID: 001093, Applied Biosystems) was used as endogenous control for data normalization. Expression levels were determined using 2^−ΔΔ*C*t^ relative quantification.

### 2.8. miR Mimics Transfections

C2C12 cells were used to perform the experimental validation of miRNA targets. The C2C12 cells were seeded one day before transfection. Next, the cells were incubated with miRNAs mimics or controls for 6 h using the transfection reagent Lipofectamine 2000 (Invitrogen). About 10 µM of mmu-miRNA-27b, mmu-miR-34b-5p, mmu-miR-760-5p miRVana mimics or miR negative control mimics (random sequences) were used for transfecting assays (Ambion, Grand Island, NY, USA, cat#4464066, cat#4464062 and cat#4464058, respectively). After 6 h, the medium was changed and DMEM supplemented with 10% of SBF, 1% of penicillin/streptomycin/glutamine was added. C2C12 cells was cultivated during 24 h and collected for analysis.

To determinate the role of these miRNAs on target genes, and consequently on adipogenesis, 3T3L1 cells was used. For this, 3T3L1 were seeded one day before transfection. The transfection with mmu-miRNA-27b, mmu-miR-34b-5p or mmu-miR-760-5p miRVana mimics were performed as previously described. After the transfection, the differentiation medium was added, and the cells were cultivated during 96 h, collected and stored at −80 °C. Triacylglycerol accumulation was also evaluated using Oil Red O method previously described.

### 2.9. Immunocytochemistry

To perform *Cebpα* and β-catenin nuclear translocation analysis, approximately 1.5 × 10^5^ cells were cultivated in a cell culture blade (Millicell^®^ EZ SLIDES, EMD Millipore Corporation, Taunton, MA, USA). After total adherence, cells were incubated with a differentiation medium (control group) or differentiation medium + guarana (150 µg/mL) (Guarana group) for 96 h (4 days). After four days, cells were fixated with paraformaldehyde, washed and incubated with PBS 0.3% triton + 5% of FBS for 1 h. Subsequently, cells were incubated with primary antibody (anti-β-catenin rabbit, and anti-Cebpα rabbit, Santa Cruz Biotechnology Inc., Santa Cruz, CA, USA) and secondary antibody (Alexa Fluor^®^ anti-rabbit, Life Technologies, Carlsbad, OR, USA) for 2 h and 1 h, respectively. DAPI (300 nM) (Sigma Aldrich, St. Louis, MO, USA) was added and incubated for 5 min. All images were obtained in fluorescence microscope (ZEISS AXIO, Carl Zeiss AG, Oberkochen, Germany) coupled to camera (AXIO CAM MRc, Carl Zeiss AG).

The analysis of β-catenin nuclear translocation and *Cebpα* were evaluated using the Image J software (National Institutes of Health, Bethesda, MD, USA). Briefly, the images were separated into three channels (RGB). For β-catenin nuclear translocation in the red channel, nuclei were selected using a freehand tool and integrated density was measured. Fifty nuclei were evaluated for each group (Control and Guarana). The *Cebpα* was quantified using the integrated density of red channel.

### 2.10. Statistical Analysis

Data are presented as mean values ± S.E.M. Gene expressions are presented as mean values ± Standard Deviation (SD). GraphPad Prism 5 was used for statistical analyses and graphics (GraphPad Software, Inc., La Jolla, CA, USA). Experimental data were analyzed by Student’s unpaired *t* test. For *Oil Red O* analysis, one-way ANOVA was used followed by the Newman-Keuls test.

## 3. Results and Discussion

It was shown that Guarana (*Paullinia cupana*) extract presents similar composition to green tea (*Camellia sinensis*) and yerba mate (*Ilex paraguariensis*), which includes a high concentration of caffeine, theobromine, theophylline, tannins, saponins, catechins, epicatechins and proanthocyanins [7]. Our data showed that the guarana used in this study presented 2.42% of flavonoids, 9.18% of total phenolics and a high caffeine content (12.4%).

**Figure 1:**
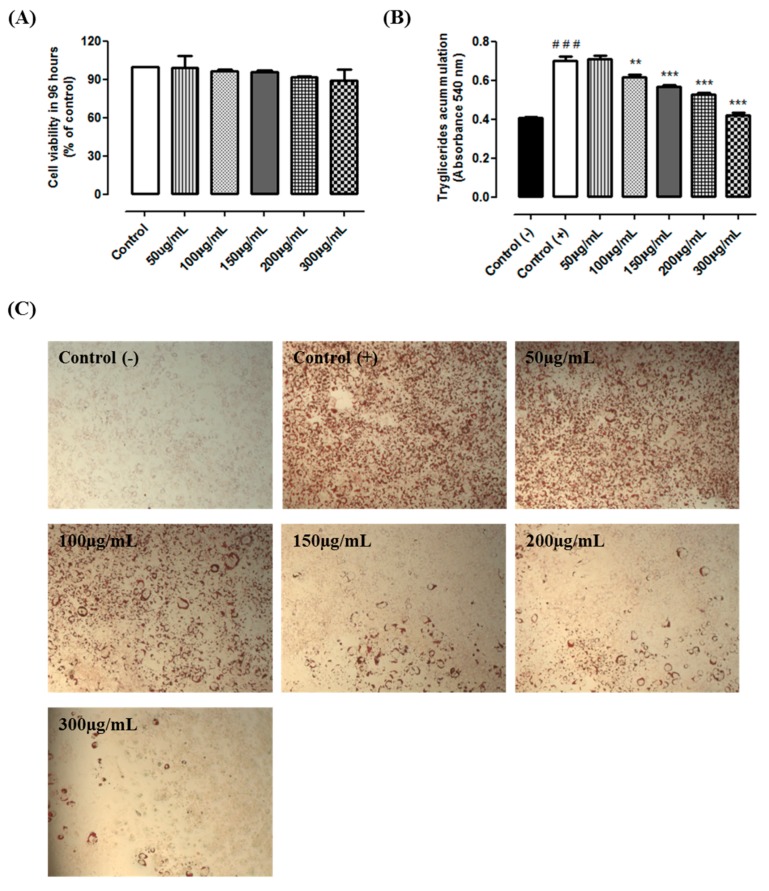
(**A**) Cell viability evaluated through MTT assay. Control—cells cultivated with DMEM; 50 µg/mL, 100 µg/mL, 150 µg/mL, 200 µg/mL and 300 µg/mL—cells cultivated with DMEM + guarana in different concentrations; (**B**) Triacylglycerol accumulation in 3T3-L1 cells according to *Oil Red O* assay. Negative control (C−)—cells cultivated with DMEM; Positive control (C+)—cells cultivated with differentiation medium (DMEM supplemented with 10% fetal bovine serum, 1% of penicillin/streptomycin/glutamine, 0.01% of dexamethasone (1 mM), 0.01% of insulin (100 UI) and 0.1% of IBMX (0.5 mM)) and 50 µg/mL, 100 µg/mL, 150 µg/mL, 200 µg/mL and 300 µg/mL—cells cultivated with differentiation medium + guarana in different concentrations. ### *p* < 0.0001 compared with C (−) and ** *p* < 0.001 and *** *p* < 0.0001 compared to C (+) group; (**C**) Illustrative images of Oil Red O in 3T3L1 cells (magnification = 200×).

The MTT results indicated that guarana is not cytotoxic in all tested conditions. Cells incubated with 50, 100, 150, 200 and 300 µg/mL during 96 h presented the viability of 98%, 96%, 95.5%, 91% and 90%, respectively (Figure 1A). The results from Oil Red O showed that cells from the positive control group presented a higher triacylglycerol accumulation when compared to the negative control group (+125%, *p* < 0.0001). Our data indicate that guarana (100, 150, 200 and 300 µg/mL) prevents the triacylglycerol accumulation (−12%, −20%, −24% and −40%, respectively, *p* < 0.0001) when compared to the positive control group (Figure 1B,C). Similarly, a recent study demonstrated that “YGD” presented similar results in 3T3L1 cells [11]. Although the effect of yerba mate on triacylglycerol accumulation has been previously shown [14], the effects of guarana have never been studied. Thereby, we are able to show that guarana has an anti-adipogenic effect preventing triacylglycerol accumulation. Recently, it was demonstrated that caffeine inhibits 3T3-L1 differentiation in a dose-dependent manner. Triacylglycerol accumulation was lower in cells treated with 1 to 5 mM caffeine. Furthermore, caffeine did not significantly affect cell viability at concentrations up to 5 mM [15]. In addition, it has been previously observed that 0.5 mM of caffeine was able to inhibit triacylglycerol accumulation in 3T3L1 cells [16]. Similar effects were observed using other cells types. Caffeine reduced lipid droplet and adipocyte levels in primary rat adipose-derived stem cells (ADSCs), and a mouse bone marrow stromal cell line (M2-10B4) [17]. Therefore, we believe that the high caffeine content might be, at least in part, responsible for the observed effect.

Adipogenesis is important to control the fat depot; however, obese subjects often present alterations in this process. It is generally believed that physical activity practice might lead to decreased body weight and percentage of body fat, improved glucose uptake and decreased fasting insulin concentrations [18]. Several studies have shown that lifestyle changes, such as healthier food intake and increased physical activity have a beneficial effect on weight loss in children andadults [19,20,21]. However, the bioactive compounds have been widely used. Studies suggest that guarana present similar effects to those observed in *Camellia sinensis* [22]. In vivo, it has been shown that *Camellia sinensis* may have beneficial effects against obesity [23,24]. Guarana exhibited a protector effect against hypertension, obesity and metabolic syndrome in elderly healthyvolunteers [8], and contributed to lower food intake in obese subjects [10]. Furthermore, the potential therapeutic role of guarana was demonstrated in atherosclerosis. The authors observed that subjects who habitually ingested guarana presented lower LDL oxidation [9]. Although the effect of guarana on obesity has been described, its role in the molecular mechanism of adipogenesis was not demonstrated.

**Figure 2:**
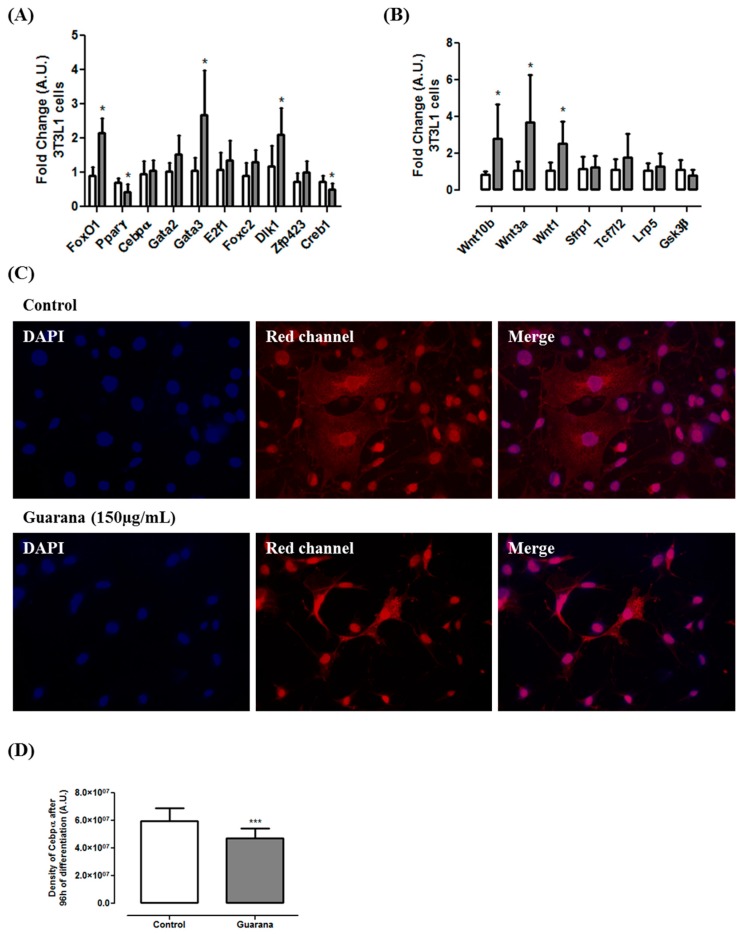
(**A**) Gene expression of *FoxO1*, *Pparγ*, *Cebpα*, *Gata2*, *Gata3*, *E2f1*, *Foxc2*, *Dlk1*, *Zfp423* and *Creb1* after 96 h of guarana treatment (150 µg/mL) in 3T3L1 cells; (**B**) Gene expression of *Wnt10b*, *Wnt3a*, *Wnt1*, *Sfrp1*, *Tcf7l2*, *Lrp5* and *Gsk3-β* after 96 h of guarana treatment (150 µg/mL) in 3T3L1 cells. White bars correspond to Control group and grey bars correspond to Guarana group. * Significative down-regulation was considered when the fold change was ≤−0.5 and significative up-regulation was considered when the fold change was ≥+2 (* *p* < 0.05); (**C**) Immunofluorescence of *Cebpα* after 96 h of incubation with differentiation medium (Control group) and differentiation medium + guarana 150 µg/mL (Guarana group) in 3T3L1 cells; (**D**) Density of *Cebpα* after 96 h of incubation with differentiation medium (Control group—white bar) and differentiation medium + guarana 150 µg/mL in 3T3L1 cells. Density was evaluated using Image J software. *** Guarana group (grey bar) *p* < 0.0001 compared to Control group (white bar).

At the molecular level, our data show that guarana was able to down-regulate the expression of pro-adipogenic genes such as *Pparγ* and *Creb1* (Figure 2A) and up-regulate the expression of anti-adipogenic genes such as *FoxO1*, *Gata3* and *Dlk1* (Figure 2A). A previous study using the YGD combination showed similar results [11], however, as far as we know, this is the first time in which the molecular mechanism of guarana has been unraveled. Caffeine (1 mM) did not affect *Cebpβ* expression but repressed *Cebpα* and *Pparγ* expression [15]. A similar effect was observed in our study, however only *Pparγ* expression was modulated by guarana. Although at mRNA level guarana does not seem to modulate *Cebpα* expression, the data from immunocytochemistry shows a significant decrease in Cebpα density (Figure 2C,D). Considering that during adipogenesis, Gata3 and Foxo1 negatively regulate Pparγ, Dlk1 is involved in the negative modulation of Cebpβ and Cebpδ, and Pparγ directly interact with Cebpα [4], we believe that guarana might exert its antiadipogenic effect, at least in part, through this pathway.

**Figure 3:**
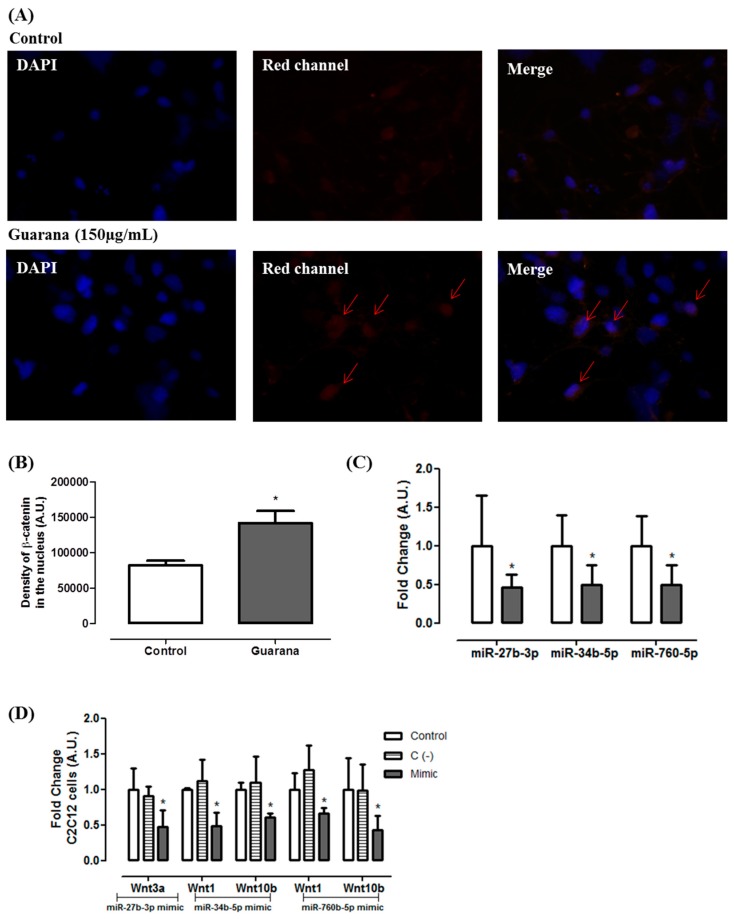
(**A**) Immunofluorescence of β-catenin after 96 h of incubation with differentiation medium (Control group) and differentiation medium + guarana150 µg/mL (Guarana group) in 3T3L1 cells; (**B**) Density of β-catenin in the nucleus after 96 h of incubation with differentiation medium (Control group—white bar) and differentiation medium + guarana 150 µg/mL in 3T3L1 cells. Density was evaluated using Image J software. ** Guarana group (grey bar) *p* < 0.001 compared to Control group (white bar); (**C**) miRNA expression of mmu-miR-27b-3p, mmu-miR-34b-5p and mmu-miR760-5p after 96 h of guarana treatment (150 µg/mL) in 3T3L1 cells. White bars correspond to Control group and grey bars correspond to Guarana group. Error bars reflect SEM. * Significative down-regulation was considered when the fold change was ≤−0.5 and significative up-regulation was considered when the fold change was ≥+2; (**D**) Gene expression of *Wnt1*, *Wnt3a* and *Wnt10b* in C2C12 cells transfected with mmu-miR-27b-3p, mmu-miR-34b-5p or mmu-miR760-5p mimics. White bars corresponding to Control, striped bars corresponding to C (−) = negative control and grey bars corresponding to mmu-miR-27b-3p, mmu-miR-34b-5p or mmu-miR760-5p mimics.

Furthermore, our results indicated that guarana induces the expression of three important Wnt pathway regulators (*Wnt1*, *Wnt3a* and *Wnt10b*) (Figure 2B). Complementarily, we found that guarana induces Wnt activation through an increase on β-catenin nuclear translocation (Figure 3A,B). It has been shown that the activation of *Wnt10b* stabilizes β-catenin in the cytoplasm favoring its translocation to the nucleus, contributing for adipogenesis inhibition [25]. However, despite Wnt10b being the primary regulator of adipogenesis, Wnt1 and Wnt3b may act synergistically for adipogenesis inhibition [26].

Considering the effects of guarana on Wnt genes, we used public microRNA target prediction databases in order to identify miRNA-gene interactions. Among the miRNAs candidates, the following were selected: mmu-miR-27b-3p (putative target—Wnt3a), mmu-miR-34b-5p (putative target—Wnt1 and Wnt10b) and mmu-miR760-5p (putative target—Wnt1 and Wnt10b). The data presented in Figure 3C shows that guarana down-regulates the expression of the three miRNAs. Our data confirmed that the miRNAs presenting the lower expression were correlated with the up-regulation of their putative target. To validate the correlation between mmu-miR-27b-3p, mmu-miR-34b-5p and mmu-miR760-5p and their targets, their mimics were transfected in C2C12 cells, and the level of their respective putative target was measured by RT-PCR.

**Figure 4:**
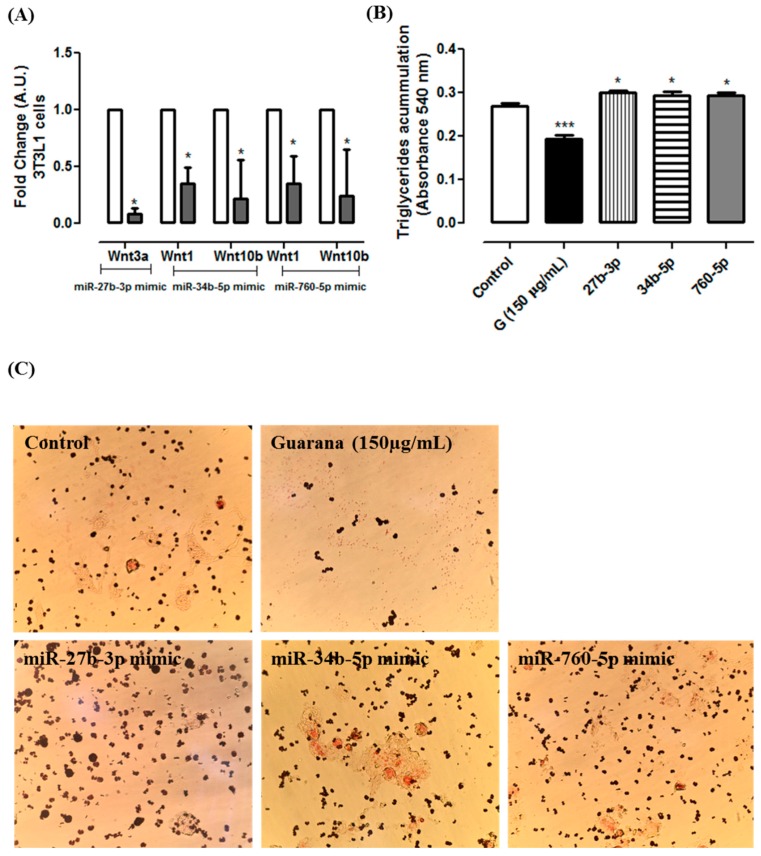
(**A**) Gene expression of *Wnt1*, *Wnt3a* and *Wnt10b* in 3T3L1 cells transfected with mmu-miR-27b-3p, mmu-miR-34b-5p or mmu-miR760-5p mimics after 96 h of differentiation. White bars corresponding to Control and grey bars corresponding to 3T3L1 transfected with mmu-miR-27b-3p, mmu-miR-34b-5p or mmu-miR760-5p mimics. * Significative down-regulation was considered when the fold change was ≤−0.5 for microRNA expressions and ≤−0.6 for mimic experiment; (**B**) Triacylglycerol accumulation in 3T3-L1 cells transfected with mmu-miR-27b-3p, mmu-miR-34b-5p or mmu-miR760-5p mimics according to Oil Red O assay. Control (C+)—cells cultivated with differentiation medium; Guarana (150 µg/mL)—cells cultivated with differentiation medium + guarana (150 µg/mL) during 96 h; 27b-3p, 34b-5p and 760-5p—cells transfected with mmu-miR-27b-3p, mmu-miR-34b-5p or mmu-miR760-5p (respectively) cultivated with differentiation medium. * *p* < 0.05 and *** *p* < 0.01 compared to Control; (**C**) Illustrative images of *Oil Red O* in 3T3L1 cells Control, Guarana (150 µg/mL) and transfected with mmu-miR-27b-3p, mmu-miR-34b-5p or mmu-miR760-5p mimics (magnification = 200×).

Figure 3D show that there was a significant *Wnt3a* repression in C2C12 cells transfected with mmu-miR-27b-3p mimic. Additionally, we found that *Wnt1* and *Wnt10b* were regulated by mmu-miR-34b-5p and mmu-miR760-5p. Furthermore, similar results were observed in 3T3L1 cells transfected with these miRNAs mimics (Figure 4A). Additionally, 3T3L1 cells transfected with mmu-miR-27b-3p, mmu-miR-34b-5p or mmu-miR760-5p mimics showed higher triglycerides accumulation when compared to 3T3L1 untransfected cells (Control group) (Figure 4B). Together, these data indicate that the miRNAs alone are capable of targeting the selected Wnt genes, and they decrease the adipogenesis.

**Figure 5:**
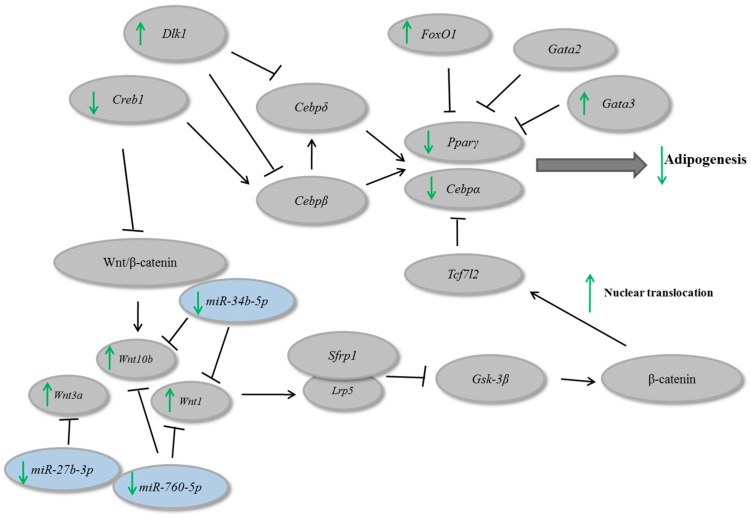
In vitro effects of guarana in gene and microRNAs involved on adipogenesis.

In a general scenario, β-catenin nuclear translocation leads to *Cebpα* and *Pparγ* repression [27]. In our study, we observed that guarana might regulate *Pparγ* and *Cebpα* through an increase on β-catenin nuclear translocation. In addition, guarana repressed *Creb1*, which is involved in adipogenesis stimulation and caused an up-regulation of *Gata3* and *Dlk1*, involved with adipogenesis inhibition. On the other hand, guarana repressed mmu-miR-27b-3p, mmu-miR-34b-5p and mmu-miR760-5p which lead to an up-regulation of their molecular targets *Wnt1*, *Wnt3a* and *Wnt10b*, contributing for adipogenesis inhibition (Figure 5). We believe that the combination of these alterations might be, at least in part, responsible for the anti-adipogenic effects of guarana.

## 4. Conclusions

This study showed that *Paullinia cupana* modulates the expression of several genes and miRNAs associated with adipogenesis, as well as an increase of β-catenin nuclear translocation, which might contribute to adipogenesis inhibition. Furthermore, we suggested that mechanistically guarana might regulate the adipogenesis thought epigenetic modulation, such as the miRNA-gene interactions described in this study. Together our data demonstrate the important role of guarana as a putative therapeutic agent in the future.

## Figures and Tables

**Table 1:** Primer sequences.

Primers	Sequence 5′→3′
*FoxO1* Fw	ACATTTCGTCCTCGAAGCAG
*FoxO1* Rv	CAGGTCATCCTGCTCTGTCA
*Pparγ* Fw	GATGGAAGACCACTCGCATT
*Pparγ* Rv	AACCATTGGGTCAGCTCTTG
*Cepbα* Fw	GGGACCATTAGCCTTGTGTG
*Cebpα* Rv	CTCTGGGATGGATCGATTGT
*Gata2* Fw	TGAAGAAGGAAGGGATCCAG
*Gata2* Rv	TGGAGAGCTCCTCGAAACAT
*Gata3* Fw	AAGTGCAAAAAGGTGCATGA
*Gata3* Rv	CAGGGATGACATGTGTCTGG
*E2f1* Fw	AGCCTAGGGATTCAGGGTGT
*E2f1* Rv	TGGATCGTGCTATTCCAATG
*Foxc2* Fw	AGGAGGCCGAGAAGAAAGTC
*Foxc2* Rv	GCTCAGCGTCTCCACCTT
*Dlk1* Fw	GGAAAAATTGGCCCCTTTAG
*Dlk1* Rv	TGGAGTCTTGGGCTAGGGTA
*Zfp423* Fw	CTTCGAGTCTCTGGCAGACC
*Zfp423* Rv	CTTGCTGGAGGGAGATGAAG
*Creb1* Fw	TTTGTCCTTGCTTTCCGAAT
*Creb1* Rv	CACTTTGGCTGGACATCTTG
*Wnt10b* Fw	TTCTCTCGGGATTTCTTGGA
*Wnt10b* Rv	CACTTCCGCTTCAGGTTTTC
*Wnt3a* Fw	GCACTAGTGTTGAGGCAATGGTCACCAG
*Wnt3a* Rv	ATACGCGTGAACGCAAAGTTCCAGGCAG
*Wnt1* Fw	GCACTAGTAGGGTTCATAGCGATCCATC
*Wnt1* Rv	ATACGCGTCAAGGAAAGGTGATAATACC
*Sfrp1* Fw	GCACTAGTATGGCATGTTGGCTGCTCTG
*Sfrp1* Rv	ATACGCGTACCTGGGAATCACTATTAAC
*Tcf7l2* Fw	CCCCTGCTTGATTGAAGTG
*Tcf7l2* Rv	GGCGGCACAAAATTAAAGAG
*Lpr5* Fw	AAGACCCTGCTTGAGGACAA
*Lpr5* Rv	TTGACCTTGTGGACCCTTTC
*Gsk3β* Fw	CTCCTCATGCTCGGATTCA
*Gsk3β* Rv	TGCAGAAGCAGCATTATTGG
